# Prospective validation of the prognostic and predictive impact of uPA/PAI-1 in early breast cancer

**DOI:** 10.1007/s10549-025-07850-z

**Published:** 2025-12-08

**Authors:** Vanessa Wieder, Julia Engel, Kathleen Eichstädt, Sandy Kaufhold, Marcus Bauer, Volker Hanf, Christoph Uleer, Susanne Peschel, Jutta John, Marleen Pöhler, Tilmann Lantzsch, Edith Weigert, Karl-Friedrich Bürrig, Jörg Buchmann, Eva Johanna Kantelhardt, Christoph Thomssen, Martina Vetter

**Affiliations:** 1 Department of Gynaecology, Martin Luther University Halle-Wittenberg, Ernst-Grube-Str. 40, 06120 Halle (Saale), Germany; 2 Institute of Pathology, Martin Luther University Halle-Wittenberg, Halle (Saale), Germany; 3 Department of Gynaecology, Nathanstift, Hospital Fürth, Fürth, Germany; 4 Present Address: Department of Gynaecology and Obstetrics, Medical University of Ulm, Ulm, Germany; 5 Gynäkologische-Onkologische Praxis Uleer, Hildesheim, Germany; 6 Present Address: Frauenärzte am Bahnhofsplatz, Hildesheim, Germany; 7 Department of Gynaecology, St. Bernward Hospital, Hildesheim, Germany; 8 Department of Gynaecology, Helios Hospital Hildesheim, Hildesheim, Germany; 9 Department of Gynaecology, Asklepios Hospital Goslar, Goslar, Germany; 10 Present Address: Department of Gynaecology and Obstretrics, Hospital Wolfenbüttel, Wolfenbüttel, Germany; 11 Department of Gynaecology, Hospital St. Elisabeth and St. Barbara, Halle (Saale), Germany; 12 Institute of Pathology, Hospital Fürth, Fürth, Germany; 13 Present Address: Gemeinschaftspraxis Pathologie Amberg, Amberg, Germany; 14 Institute of Pathology Hildesheim, Hildesheim, Germany; 15 Institute of Pathology, Hospital Martha-Maria, Halle (Saale), Germany; 16 Institute of Epidemiology, Biometry and Informatics, Martin Luther University Halle-Wittenberg, Halle (Saale), Germany

**Keywords:** uPA/PAI-1, Early breast cancer, Prognostic factor, Predictive factor

## Abstract

**Introduction:**

An emerging challenge in early breast cancer (eBC) is improving risk assessment through the use of biomarkers. Clinical guidelines have recommended urokinase-type plasminogen activator (uPA) and its inhibitor PAI-1 for risk evaluation. This study aimed to validate the prognostic and predictive impact of uPA/PAI-1.

**Patients and methods:**

From a prospective cohort of 1270 patients (PiA-study, Prognostic assessment in routine Application, NCT01592825), concentrations of uPA and PAI-1 were determined in fresh tumour tissue (*n* = 813) by ELISA (FEMTELLE®; LOXO Diagnostics). The uPA/PAI-1 status was defined as low if both uPA and PAI-1 levels were low and as high if one or both were elevated. Primary objectives were the distribution of the uPA/PAI-1 status and its association with clinical/histopathological parameters. Secondary objectives were the association of the uPA/PAI-1 status with recurrence-free interval (RFI), overall survival (OS), and benefit from adjuvant chemotherapy.

**Results:**

A low uPA/PAI-1 status was observed in 37.6% (306 of 813) of the entire cohort and in 47.9% (181 of 378) of those classified as intermediate-risk patients (≥ 35yrs, ≤ pN1, G2, sHR positive/HER2 negative). A low uPA/PAI-1 status was associated with parameters that predict a favourable prognosis. Overall, 96.7% (95% CI 94.5–98.9) of patients with a low uPA/PAI-1-status remained recurrence-free at five years and 87.2% (95% CI 84.1–90.3) with a high uPA/PAI-1 status even after adjustment to tumour size, nodal status, grading, steroid hormone receptor (sHR) status and HER2 status (adjusted HR 2.6, 95% CI 1.29–5.23). Among intermediate-risk patients without chemotherapy (*n* = 197), the prognostic value was even more pronounced (HR 10.10, 95% CI 1.13–16.12). Similar results were observed for OS. Only patients with a high uPA/PAI-1 status appeared to benefit from chemotherapy (adjusted HR 0.28, 95% CI 0.07-1.12, *p* = 0.07).

**Conclusion:**

This prospective analysis confirms the uPA/PAI-1 status as an independent prognostic factor and suggests a predictive impact considering benefit from chemotherapy.

**Supplementary Information:**

The online version contains supplementary material available at 10.1007/s10549-025-07850-z.

## Introduction

Breast cancer (BC) is a highly heterogeneous disease that necessitates precise analysis of individual biology and recurrence risk to define appropriate therapy. For this purpose, clinical and histopathological factors are employed. In particular, for patients with an intermediate risk of recurrence, biomarkers can aid in refining treatment decisions. One such biomarker may be the combination of the invasion factor urokinase-type plasminogen activator (uPA) and its inhibitor, plasminogen activator inhibitor-1 (PAI-1). As prognostic markers for node-negative early breast cancer (eBC), uPA and PAI-1 were recommended in Europe since 2005 [1] as well as by the American Society of Clinical Oncology (ASCO) [2]. Initially, several studies emphasized the role of uPA in extracellular matrix degradation by promoting cell surface-associated proteolysis, followed by the detachment of cancer cells from their microenvironment, a prerequisite for metastasis formation. Additionally, upon binding to the uPA receptor (uPAR), the MAPK/ERK signaling pathway is activated, inducing proliferation, migration, and cell adhesion [3]. Nearly 40 years ago, Duffy et al. discovered that eBC patients with high uPA proteolytic enzyme activity in their tumours had a significantly shorter disease-free interval, suggesting uPA as a prognostic marker [4]. Subsequently, Jänicke et al. demonstrated a strong correlation between elevated uPA antigen levels in the primary tumour and a poor prognosis in eBC patients [5]. PAI-1 inhibits uPA, thereby reducing proteolytic activity; the PAI-1/uPA/uPAR complex is internalized and degraded [6]. However, PAI-1 also promotes tumour cell adhesion and invasion through surface receptors such as integrins, as well as angiogenesis via VEGF, crucial for tumour growth and metastasis [7]. Jänicke et al. [8] showed that the combination of uPA and PAI-1 levels, referred to as uPA/PAI-1 status, provided a more accurate prediction of disease-free survival than either factor alone, as confirmed by Foekens et al. [9] and Harbeck et al. [10]. Low expression of both proteins is associated with superior survival [11].

In the Chemo-N0 trial, the first prospective trial focusing on biomarker-guided therapy for eBC patients, the long-term prognostic significance of the uPA/PAI-1 status was validated. The results suggest that node-negative patients with high uPA/PAI-1 status benefit from adjuvant chemotherapy [12]. The prospective NNBC 3-Europe trial was conducted to further validate the prognostic significance of uPA/PAI-1 in the context of modern therapy regimens [13]. In contrast to most previous studies, which focused on selected clinical trial populations, this study prospectively evaluated uPA/PAI-1 status as a biomarker in an unselected early breast cancer cohort drawn from daily routine practice.

## Methods and material

### Study design

In total, 1270 eBC patients were consecutively enrolled from five certified Breast Cancer Centers in Germany (2009–2011). This prospective cohort, registered as the “PiA-study” (Prognostic assessment in routine Application; NCT01592825), was designed to validate the prognostic impact of uPA/PAI-1 in daily routine in accordance with the REMARK criteria [14]. Patients were enrolled based on the following criteria: female, ≥ 18 years, invasive eBC and no second cancer [15]. Patients who received neoadjuvant chemotherapy (NACT) were not included in this analysis due to the lack of available fresh frozen tissue. Tumours were staged and graded according to guidelines. The expression of estrogen receptor and progesterone receptor were combined as steroid hormone receptor (sHR) status and considered positive if at least one receptor exhibited ≥ 1% positivity. The human epidermal growth factor receptor 2 (HER2) status was determined according to the ASCO/CAP recommendations [16]. All diagnostic and therapeutic procedures were applied in compliance with the annually updated German Recommendations of the AGO (Arbeitsgemeinschaft Gynäkologische Onkologie, Breast Committee of the Gynecological Oncology Working Group) valid at the respective time of diagnosis [17]. The clinically intermediate-risk group was defined as patients aged ≥ 35 years with ≤ pN1, G2, sHR positive/HER2 negative tumours, according to the uPA/PAI-1 algorithm from the randomized NNBC 3-Europe trial [18]. In line with MINDACT and RxPONDER trials [19, 20], patients with limited nodal involvement (pN1) were included, as such status does not invariably indicate high risk or a uniform chemotherapy benefit. To ensure a homogeneous cohort and minimize confounding from endocrine therapy, we also performed analyses that were restricted to sHR positive/HER2 negative patients.

### Tumour preparation and uPA and PAI-1 determination

Tissues from surgical primary specimens were collected by the pathologist at each center, immediately snap-frozen in liquid nitrogen, and shipped on dry ice for centralized testing. Tissue samples (30–400 mg) were pulverized under liquid nitrogen, and protein extraction was performed overnight at 4 °C using 300–1000 µL Tris extraction buffer (50-mM Tris pH 8.5, 138-mM NaCl, 2.7-mM KCl with 1% Triton X-100), according to the standard protocol [21, 22]. Following centrifugation at 13,000×*g* for 1 h at 4 °C, the cytosolic supernatants were used to determine uPA and PAI-1 antigen concentrations via enzyme-linked immunosorbent assay (FEMTELLE®, LOXO Diagnostics GmbH Dossenheim, originally American Diagnostica, USA), and measured as ng analyte per mg total protein. The total protein concentration was assessed using BCA Protein Assay (Pierce™, Rockford, IL, USA). Internal quality assurance was implemented for within-assay and between-assay variability [23].

### Risk assessment by uPA/PAI-1 status

uPA/PAI-1 status was defined as low if  both, uPA and PAI-1 concentrations were below the established cut-off values (uPA ≥ 3 ng/mg total protein, PAI-1 ≥ 14 ng/mg total protein) [24, 25], high uPA/PAI-1 status was assigned if at least one of the protein measurements was elevated. As a consecutive cohort study, the implementation of the uPA/PAI-1 status in treatment decisions was not mandatory. Thus, in the intermediate-risk group, over one-third of patients with high uPA/PAI-1 did not receive chemotherapy. In such cases, the decision was influenced by a number of factors, including comorbidities and patient preference. And patients with low uPA/PAI-1 status received adjuvant chemotherapy in one-third of the cases, often due to tumour size or increased safety concerns.

### Endpoints and statistical analysis

As first objective, we determined the proportion of tumours with a low or high uPA/PAI-1 status and its association with clinical and histopathological characteristics in the total cohort as well as in subgroups, using binary logistic regression (odds ratio, OR) and Pearson *χ*^2^ test. As second objective, we assessed its prognostic and predictive impact in the total cohort and for patients with an intermediate risk of recurrence. The endpoints were recurrence-free interval (RFI), defined as local invasive recurrence, distant recurrence, or death from BC, and overall survival (OS), defined as death from any or unknown cause, according to the standardized definitions of efficacy endpoints (STEEP) criteria [26]. Accordingly, the term “recurrence” corresponds to RFI-events. Univariable analyses of RFI and OS were performed using Kaplan–Meier estimates with the log-rank test, and multivariable analyses were conducted using Cox proportional hazards models, including pre-specified confounding factors. Predictive analyses were carried out in selected treatment groups to evaluate the benefit of chemotherapy. The test for interaction was performed using multivariable Cox analysis, incorporating chemotherapy and the uPA/PAI-1 status as a combined variable [27]. All *p* values were two-sided, with values of < 0.05 considered statistically significant. Statistical analyses were conducted using SPSS 28 (IBM Corp., Armonk, NY, USA). Forest plots were generated using Python 3.13.2.

## Results

### Patient’s and tumour characteristics of the uPA/PAI-1 cohort

**Fig. 1:**
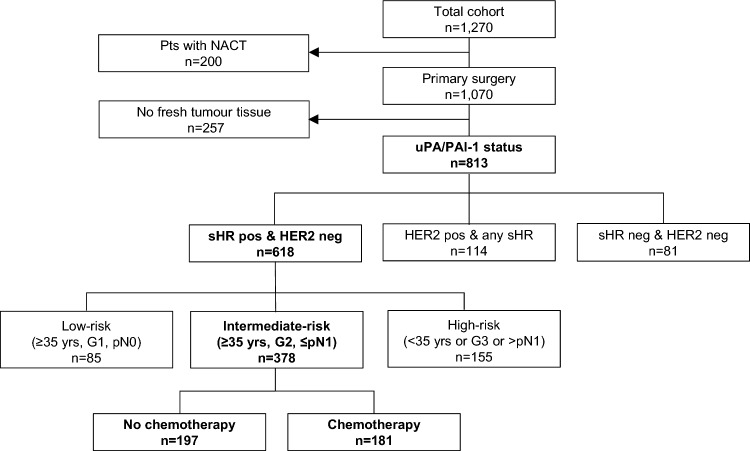
Patients of the PiA cohort (*n* = 1,270) and groups that were used for multivariable uPA/PAI-1 analysis (*n* = 813). *Pts* patients, *uPA* urokinase-type plasminogen activator, *PAI-1* plasminogen activator inhibitor type 1, *sHR* steroid hormone receptor, *HER2* human epidermal growth factor receptor 2, *NACT* neoadjuvant chemotherapy

All patients with available fresh frozen tissue were considered for determination of the uPA and PAI-1 concentrations (*n* = 813) (Fig. 1). The median age was 56 years (22–90 years), with three-quarters of patients being older than 50 years. The majority of tumours (76.0%) were sHR positive and simultaneously HER2 negative (hereinafter referred to as sHR positive/HER2 negative), 14,0% were HER2 positive regardless of sHR status, and 10.0% were classified as triple-negative breast cancer (TNBC). When comparing the uPA/PAI-1-cohort with the entire study cohort, the uPA/PAI-1-cohort had 3.8% more sHR positive tumours (85.5% vs. 81.7%) and 2.6% fewer HER2-positive tumours (14,0% vs. 16.5%) (*χ*^2^ test; *p* < 0.05), reflecting the use of neoadjuvant therapy with less available tumour tissue in sHR-negative tumours (Supplementary Table S1).

### Proportion of patients with low and high uPA/PAI-1 status and its association with clinical and histopathological parameters

Overall, the uPA/PAI-1 status could be determined for 64.0% (*n* = 813) of the total cohort (*n* = 1,270). Among these patients, 37.6% (306 of 813) had a low uPA/PAI-1 status and 62.4% (507 of 813) had a high uPA/PAI-1 status. In the node-negative subgroup, a similar proportion was observed (39% and 61%, respectively) (Suppplementary Table S2).

**Table 1:**
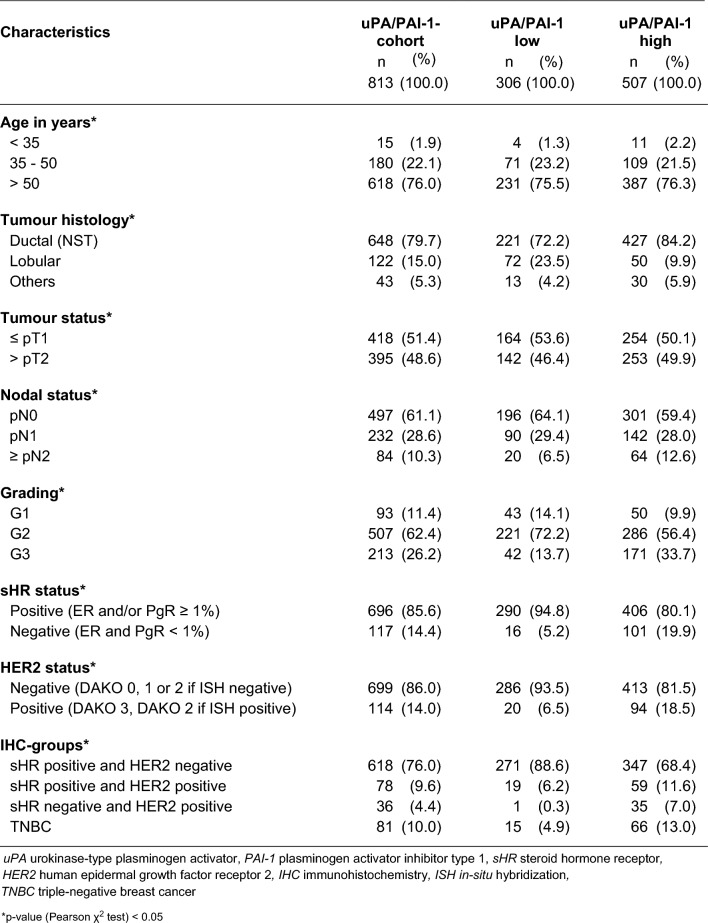
Prevalence of low and high uPA/PAI-1 status in selected groups

Tumours with a high uPA/PAI-1 status were associated with advanced lymph node involvement (12.6% vs. 6.5%; OR = 2.07, 95% CI 1.224–3.488, (Table 1, Supplementary Tables S2, S3) and poorer tumour grading (33.7% vs. 13.7%; OR = 3.50, 95% CI 2.063–5.944). Conversely, tumours larger than 5 cm more frequently exhibited a low uPA/PAI-1 status (OR = 2.21, 95% CI 1.087–4.503). Tumours with a low uPA/PAI-1 status were more likely to be sHR-positive (94.8% vs. 80.1%; OR = 4.57, 95% CI 2.939–7.897) and HER2 negative (93.5% vs. 81.5%; OR = 3.21, 95% CI 1.937–5.328). Among sHR positive/HER2 negative tumours (*n* = 618), 43.9% had a low uPA/PAI-1 status. In the intermediate-risk group (*n* = 378), the proportion of patients with a low uPA/PAI-1 status (47.9%, 181 of 378) was higher than in the total cohort (Table 2). The median follow-up was 60.3 months (1–129) and survival analyses were censored at 60-months obervation time.

**Table 2:** Distribution of uPA/PAI-1 low-risk tumours in more homogeneous subgroups

Patient groups	uPA/PAI-1 low
	*n* (%)
Total uPA/PAI-1-cohort (*n* = 813)	306 (37.6)
sHR positive and HER2 negative (*n* = 618)	271 (43.9)
Intermediate-risk group* (*n* = 378)	181 (47.9)
Intermediate-risk group*, no CT (*n* = 197)	120 (60.9)

*uPA* urokinase-type plasminogen activator, *PAI-1* plasminogen activator inhibitor type 1, *sHR* steroid hormone receptor, *HER2* human epidermal growth factor receptor 2, *CT* chemotherapy

* ≥ 35yrs, ≤ pN1, G2, sHR positive/HER2 negative

### Prognostic impact of uPA/PAI-1 status

**Fig. 2:**
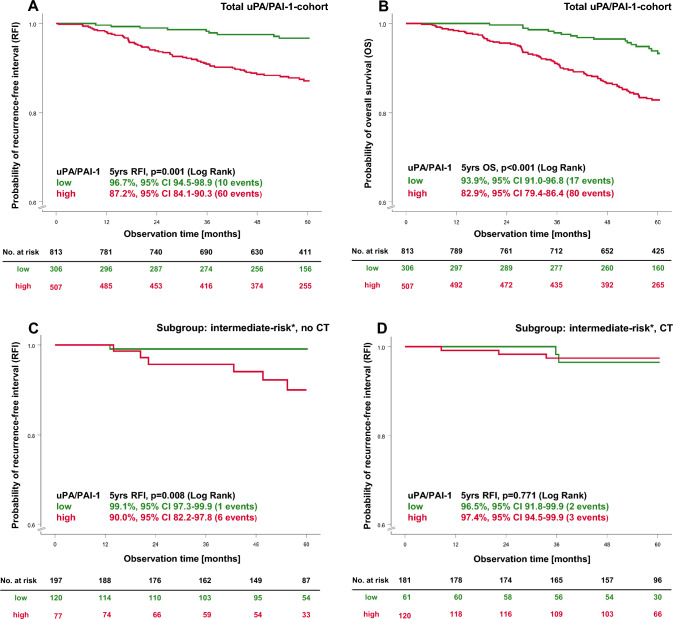
Survival estimates for uPA/PAI-1 status with regard to 5-year RFI and OS in the total uPA/PAI-1-cohort (**A**, **B**) and in the intermediate-risk group (*n* = 378), without adjuvant chemotherapy (**C**), and treated with chemotherapy (**D**). The tables present the effective sample size for each interval (No. at risk). *uPA* urokinase-type plasminogen activator, *PAI-1* plasminogen activator inhibitor type 1, *sHR* steroid hormone receptor, *HER2* human epidermal growth factor receptor 2, *CT* chemotherapy. * ≥ 35yrs, ≤ pN1, G2, sHR positive/HER2 negative

**Fig. 3:**
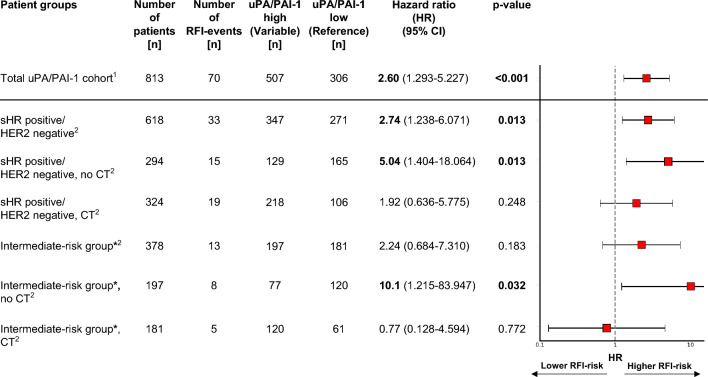
Impact of uPA/PAI-1 status with regard to 5-year RFI in the total cohort and in selected subgroups. *uPA* urokinase-type plasminogen activator, *PAI-1* plasminogen activator inhibitor type 1, *sHR* steroid hormone receptor, *HER2* human epidermal growth factor receptor 2, *CT* chemotherapy, *CI* confidence interval. ^1^Adjusted to tumour size, nodal status, grading, sHR status, and HER2 status. ^2^Univariable HR in this selected subgroup. * ≥ 35 yrs, ≤ pN1, G2, sHR positive/HER2 negative

Overall, 96.7% of patients with a low uPA/PAI-1 status were recurrence free after five years of follow-up, compared with 87.2% of those with a high uPA/PAI-1 status (HR 3.86, 95% CI 1.977–7.544) (Fig. 2A). Even after adjustment to tumour size, sHR status, HER2 status, grading, and nodal status, patients with a high uPA/PAI-1 status had significantly higher risk of recurrence compared to those with a low uPA/PAI-1 status (HR 2.60, 95% CI 1.293–5.227) (Table 3, Fig. 3). A significant difference was also observed for OS: 93.9% of patients with a low uPA/PAI-1 status were still alive after 5 years, compared with 82.9% with a high uPA/PAI-1 status (Fig. 2B) (adjusted HR 2.38, 95% CI 1.359–4.156, Supplementary Table S4). The prognostic impact of the uPA/PAI-1 status with respect to both RFI and OS was consistent across all clinically relevant subgroups (Fig. 3, Supplementary Fig. S3, Supplementary Tables S4–S6). Among patients with sHR-positive/HER2-negative tumours and a low uPA/PAI-1 status, 97.1% remained recurrence free, compared to 91.5% with a high uPA/PAI-1 status (HR 2.74, 95% CI 1.238–6.071, *p* = 0.013) (Fig. 3, Supplementary Fig. S1A). A similar pattern was observed for OS (Supplementary Fig. S1B; Tables S5, S6). Node-negative patients with a high uPA/PAI-1 status had a significant higher risk for an RFI event compared to patients with low uPA/PAI-1 status (HR 3.95, 95% CI 1.369-11.376) (Supplementary Fig. S3).

**Table 3:** Univariable and multivariable analyses with regard to 5-year RFI for selected characteristics

Characteristics			Recurrence-free interval, 5 years (70 events)			
			Univariable analysis		Multivariable analysis	
	Sample size	Events	Hazard ratio	95% CI	Hazard ratio	95% CI
uPA/PAI-1 status						
Low	306	10	1	–	1	–
High	507	60	**3.86**	1.977–7.544	2.60*	1.293–5.227
Age in years						
< 35	15	2	1.14	–	–	–
35–50	180	17	1.67	0.407–6.872	–	–
> 50	618	51	1	0.655–1.970	–	–
Tumour size in cm						
≤ 2	418	20	1	–	1	–
> 2	395	50	**2.86**	1.701–4.811	1.65	0.948–2.886
Nodal status						
pN0	497	28	1	–	–	–
pN1	232	17	1.30	0.709–2.367	–	–
≥ pN2	84	25	**6.31**	3.669–10.857	–	–
Nodal status						
pN0 and pN1	729	45	1	–	1	–
≥ pN2	84	25	**5.77**	3.526–9.426	**3.82**	2.255–6.480
Grading						
G1	93	1	1	–	1	–
G2	507	35	6.76	0.920–49.148	5.04	0.685–37.064
G3	213	34	**16.71**	2.287–122.054	6.63	0.875–50.213
IHC groups						
sHR positive/HER2 negative	618	34	1	–	1	–
HER2 positive any sHR	114	16	**2.60**	1.434–4.711	1.42	0.756–2.666
TNBC	81	20	**5.05**	2.905–8.788	**2.76**	1.482–5.128

*uPA* urokinase-type plasminogen activator, *PAI-1* plasminogen activator inhibitor type 1, *sHR* steroid hormone receptor, *HER2* human epidermal growth factor receptor 2*, IHC i*mmunohistochemistry*, TNBC* triple-negative breast cancer, *CI* confidence interval

*Adjusted to tumour size, nodal status, grading and IHC types. Bold: *p* value < 0.05

### Predictive impact of uPA/PAI-1 status for sHR positive/HER2 negative patients

Nearly half of the sHR positive/HER2 negative patients (*n* = 294) received endocrine treatment only. Among those who did not receive chemotherapy, patients with a low uPA/PAI-1 status (*n* = 165) had a 98.1% probability to remain free of recurrences, compared with 89.9% for those with a high uPA/PAI-1 status (*n* = 129) (Supplementary Fig. S1C). This indicated a fivefold higher probability of recurrence with a high uPA/PAI-1 status (HR 5.04, 95% CI 1.404–18.064) (Fig. 3). A similar effect was seen for OS (Supplementary Fig. S1E, Supplementary Table S6). Among patients who had received chemotherapy (*n* = 324), no significant difference in RFI or OS was observed between those with a low (*n* = 106) and high (*n* = 218) uPA/PAI-1 status (Fig. 3; Supplementary Fig. S1D, F; Table S6).

**Fig. 4:**
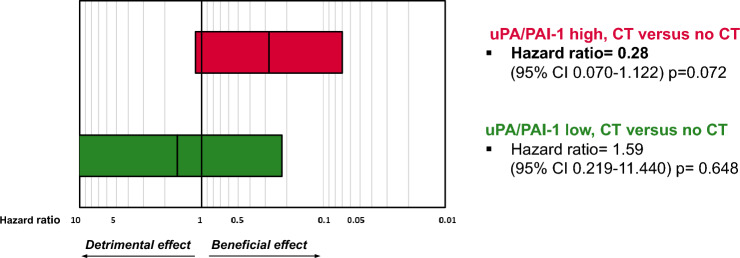
Effects of therapy with regard to uPA/PAI-1 high (red bar) and uPA/PAI-1 low (green bar) are illustrated as adjusted hazard ratios for 5-year RFI in the intermediate-risk group. *uPA* urokinase-type plasminogen activator, *PAI-1* plasminogen activator inhibitor type 1, *CT* chemotherapy*, CI* confidence interval. intermediate–risk group: ≥ 35yrs, ≤ pN1, G2, sHR positive/HER2 negative

Among these patients with sHR positive/HER2 negative tumours and an intermediate risk of recurrence who did not receive adjuvant chemotherapy, a high uPA/PAI-1 status (*n* = 77) was associated with a tenfold higher risk of recurrence compared with patients who had a low uPA/PAI-1 status (*n* = 120; HR 10.10, 95% CI 1.215–83.947) (Fig. 3), corresponding to absolute risks of recurrence of 10.0% and 0.9%, respectively (Fig. 2C, Supplementary Table S5). Even after adjustment for tumour size, the prognostic impact remained strong (HR 9.90, 95% CI 1.188–82.5370).Again, among patients who received chemotherapy (*n* = 181), no significant impact of the uPA/PAI-1 status on RFI was observed (Fig. 2D). A similar trend was seen for OS (Supplementary Fig. S3C, D). In summary, a trend can be recognized suggesting that patients with a high uPA/PAI-1 status may have benefited from adjuvant chemotherapy (HR 0.28, 95% CI 0.070–1.122, *p* value 0.072, test for interaction *p* = 0.078) (Fig. 4). No detectable effect of adjuvant chemotherapy on RFI was found in patients with a low uPA/PAI-1 status.

## Discussion

The aim of our prospective study was to assess the real-world prevalence of the uPA/PAI-1 status in a multicenter prospective cohort (*n* = 1270) of eBC patients and to confirm its prognostic and predictive impact.

In the pivotal exploratory studies, evaluation of uPA/PAI-1 classified at least 56% of node-negative patients as having low uPA/PAI-1 [11, 28–30]. By contrast, we observed a low uPA/PAI-1 status in only 40% of node-negative patients, consistent with findings from the Chemo-N0 [12] and NNBC 3-Europe [13] clinical trials. While patient selection may have influenced the results in these trials, such selection bias was minimized in the present cohort analysis. We also confirmed the established cut-off values in our data (data not shown), ruling out a shift in cut-offs as an explanation for the lower-than-expected prevalence of low uPA/PAI-1. In summary, based on the present data, a proportion of 40% low uPA/PAI-1 status in eBC patients reflects the real-world prevalence.

A low uPA/PAI-1 status was associated with clinical and histopathological characteristics known to predict a more favourable course of disease. The observed enrichment of low uPA/PAI-1 status in larger tumours (> 5 cm) likely reflects a non-aggressive tumour biology in these specimens. This might explained by a selection effect since patients with large, aggressive tumours frequently present with metastases (advanced BC) at time of diagnosis were not included in our uPA/PAI-1 study as a cohort of eBC.

The prognostic impact of uPA/PAI-1 was originally established in the unicenter study by Jänicke et al. [29], later validated in the clinical trial Chemo-N0 [12], and further confirmed in a large pooled analysis by Look et al. [31]. At that time, most node-negative patients did not receive adjuvant chemotherapy. This prospective cohort analysis confirms that uPA/PAI-1 status retains a strong and independent prognostic impact in eBC patients, even after adjustment for clinical and histopathological factors and the application of contemporary adjuvant therapies. Notably, we observed a shift to better clinical outcome compared with earlier studies: In the initial investigations of untreated patients, those with high uPA/PAI-1 had a 5-year recurrence rate of 35% [11], whereas in our analysis, only 13% of high-risk patients experienced recurrence over the same period. The treatment effect was particularly pronounced in the intermediate-risk group receiving adjuvant chemotherapy, in whom the prognostic difference was completely abolished, unlike in patients who did not receive treatment.

The prognostic value of uPA/PAI-1 was consistently observed across all age groups, tumour sizes, and grades of differentiation, underscoring its robustness as a biomarker in eBC. This prognostic relevance was particularly pronounced in tumours with more favourable biological characteristics, including sHR-positive tumours, HER2-negative tumours, and those with less lymph node involvement.

In patients with sHR positive/HER2 negative tumours (*n* = 618), we observed an absolute increase of 6% in the proportion of patients with a low uPA/PAI-1 status compared with the entire cohort (44% vs. 38%, respectively), with a still notably low risk of recurrence (7 of 271; HR 2.74). Harbeck et al. previously reported that the proportion of a low uPA/PAI-1 status was higher in G2 tumours than in the entire study cohort of the NNBC 3-Europe trial [32]. Based on results from the Chemo-N0 trial and routine clinical practice, an intermediate-risk group was defined, consisting of patients aged 35 years and older, with moderately differentiated tumours (G2) and no lymph node involvement. This clinical definition served as the basis for risk stratification and treatment in the NNBC 3-Europe trial using uPA/PAI-1 [18]. In the present analysis, about 50% of patients with clinically intermediate-risk had a low uPA/PAI-1 status. In patients with 1–3 involved lymph nodes, also 48% had a low uPA/PAI-1 status. Similarly, in a retrospective unicenter study, Kolben et al. analyzed a comparable subgroup using uPA/PAI-1 status, and again half of the patients were classified as low risk [33]. In our analysis, patients within the clinically intermediate-risk group who had a low uPA/PAI-1 status showed a favourable 5-year recurrence-free rate of 99.1%, even without chemotherapy (comprising 60.9% of these patients), with an HR of 10.1 (Fig. 3, Supplementary Table S5). In summary, an increased prognostic impact of uPA/PAI-1 was observed in clinically more defined subgroups.

To position our findings relative to existing risk assessment tools, we compared the performance of uPA/PAI-1 with gene expression profiles (GEPs), such as Oncotype DX Breast Recurrence Score®, EndoPredict®, Prosigna®, and MammaPrint® (Supplementary Fig. S4). As observed in our dataset, a similar distribution of low and high risk in patients who did not receive chemotherapy was seen with EndoPredict® (EPclin Risk Score): 95.3% of patients classified as low risk (37% of the total cohort) were free from distant recurrences after 10 years, compared with 89.1% in the high-risk group [34]. In the MINDACT trial, MammaPrint® reclassified 46% of patients with a high *clinical* risk to low *genomic* risk in sHR-positive/HER2-negative tumours, resulting in a 5-year disease-free survival rate of 90.3% without chemotherapy, compared with 85.7% in the high genomic risk group [19]. Using Oncotype DX®, three clinical trials showed that postmenopausal patients with an intermediate recurrence score (RS 11–25) and sHR-positive/HER2 negative tumours achieved excellent outcomes with endocrine therapy alone, even in those with one to three involved lymph nodes, classifying a large proportion of patients as low risk [20, 35, 36]. Prosigna® classified 56% of postmenopausal patients as low risk, with a 10-year freedom from distant recurrence rate of 95.0%, compared with 82.2% in the high-risk group (Supplementary Fig. S4). In a within-patient comparison, Sestak et al. found that the signatures providing the most useful prognostic information in postmenopausal, node-negative patients with ER-positive/HER2 negative tumours were Prosigna® (ROR; HR 2.56) and EndoPredict® (EPclin; HR 2.14). The Breast Cancer Index® also showed strong performance in their study [37]. Bechthold et al. [38] confirmed these findings using data from the PiA study cohort. In our present analysis of patients with sHR positive/HER2 negative tumours, the uPA/PAI-1 status demonstrated a prognostic impact comparable to the aforementioned GEPs (HR 2.7).

Otherwise usable prognostic factors are currently not applicable to sHR positive tumours. For example, tumour-infiltrating lymphocytes (TILs) have shown no prognostic impact in this subgroup, as observed in our cohort by Schüler et al. [39]. By contrast, TILs demonstrated significant prognostic relevance in TNBC, with a molecular subtype that present low amounts of TILs, which are associated with poorer survival [40]. These findings highlight the importance of uPA/PAI-1 as a prognostic marker, particularly in endocrine-sensitive tumours, as reported in the ASCO-guidelines by André et al. [41].

We further analyzed the predictive value of the uPA/PAI-1 status in patients with an intermediate risk of recurrence considering the effect of adjuvant chemotherapy. In patients with a high uPA/PAI-1 status, a possible benefit from adjuvant chemotherapy was indicated, while those with a low status showed no benefit, suggesting an option to avoid chemotherapy. This aligns with findings by Harbeck et al., who found that chemotherapy reduced the risk of relapse in patients with a high uPA/PAI-1 status, particularly among those with zero to three affected lymph nodes [27]. Analysis of this subgroup revealed a significant interaction between the uPA/PAI-1 status and chemotherapy efficacy, closely mirroring our results. Similarly, the 10-year follow-up of the Chemo-N0 trial showed that nearly half of lymph node-negative patients could forgo chemotherapy, while high-risk patients derived a significant benefit [12].

To further refine the utility of uPA/PAI-1, Engel et al. demonstrated in an exploratory analysis that high *NOTCH1* mRNA expression is strongly associated with a high uPA/PAI-1 status (OR 1.90), suggesting a link between Notch signaling and the regulation of tumour invasiveness. Patients with both high *NOTCH1* mRNA-expression and a high uPA/PAI-1 status had the poorest prognosis, with a significantly increased risk of recurrence (adjusted HR 3.70) and reduced OS [42] and appeared to be resistant to adjuvant chemotherapy.

## Strengths and limitations

The PiA study is a prospective multicentre cohort study that provides valid insights into the natural distribution of biomarkers. Standardized and annually updated therapy algorithms were applied consistently, in accordance with the German AGO recommendations valid at the respective time. For all prognostic and predictive analyses, strict endpoint criteria were used, focusing solely on disease-related events (RFI) and OS. A limitation of the study is that, although cohort design reduce recruitment bias, treatment decisions were guided by clinical guidelines rather than randomization, which may affect prognostic analyses. Due to the low number of events, detailed subgroup analyses were limited. Currently, uPA/PAI-1 testing is limited by the requirement for fresh frozen tissue. While uPA/PAI-1 testing can be performed using core needle biopsies [43], in our study no fresh frozen tissue was available from patients who received core needle biopsy and subsequent NACT, and consequently, NACT patients were not included in our study.

## Conclusion

The present data confirm that the prognostic and predictive impact of the uPA/PAI-1 status for eBC patients has been consistently reproduced for over four decades. Our findings underscore its utility in identifying patients who are most likely to benefit from adjuvant chemotherapy. These results support current guideline recommendations, which advocate incorporating biology-based prognostic factors when clinical risk estimation alone is insufficient.

## Supplementary Information

Below is the link to the electronic supplementary material.

Supplementary file1 (PDF 697 KB)

## Data Availability

Raw data are available on request to the corresponding author.
