# Supplementary material for: Prospective validation of the prognostic and predictive impact of uPA/PAI-1 in early breast cancer
Source: Breast Cancer Res Treat. 2025 Dec 8;215(1):31. doi: 10.1007/s10549-025-07850-z (PMC12686101; doi:10.1007/s10549-025-07850-z)
Supplement: Supplementary file 1 — Supplementary file1 (PDF 697 KB) [file 10549_2025_7850_MOESM1_ESM.pdf]

Supplement

Prognostic and predictive impact of uPA/PAI-1 in early breast cancer

Vanessa Wieder, Julia Engel, Kathleen Eichstädt, Sandy Kaufhold, Marcus Bauer, Volker Hanf, Christoph Uleer, Susanne Peschel, Jutta John, Marleen Pöhler, Tilmann Lantzsch, Edith Weigert, Karl-Friedrich Bürrig, Jörg Buchmann, Eva Johanna Kantelhardt, Christoph Thomssen, Martina Vetter

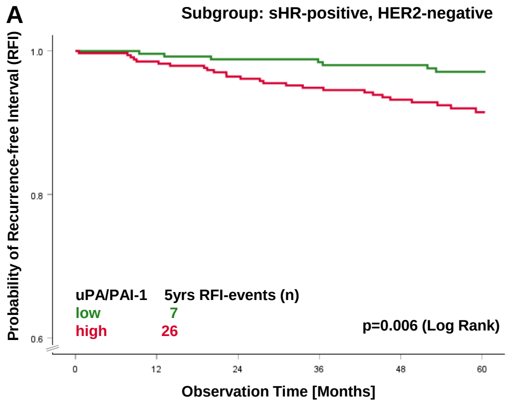

No. at risk	618	595	572	538	495	302
low	271	262	254	243	229	134
high	347	333	318	295	266	168

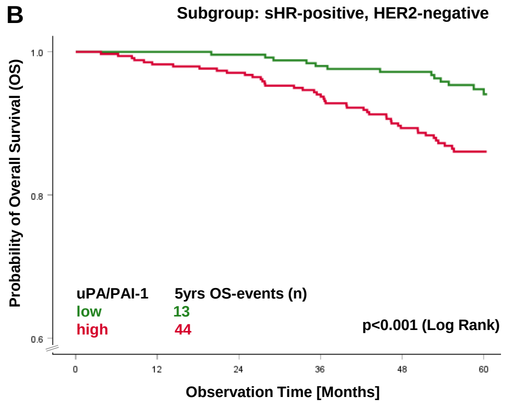

No. at risk	618	599	583	551	508	307
low	271	263	256	245	232	136
high	347	336	327	306	276	171

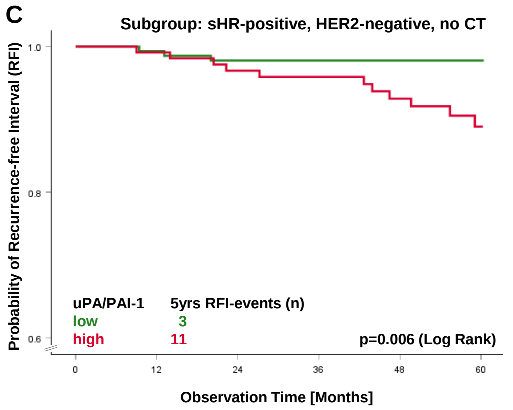

No. at risk	294	282	266	247	225	136
low	165	158	152	144	134	80
high	129	124	114	103	91	56

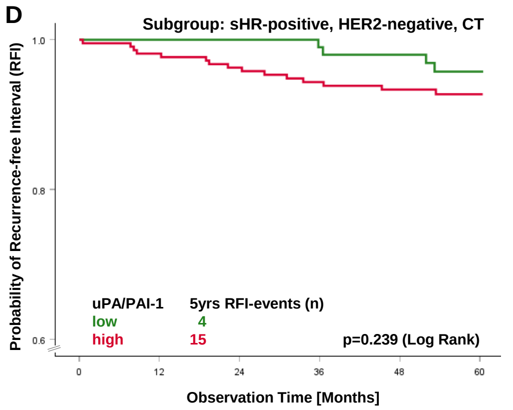

No. at risk	324	314	306	291	270	165
low	106	104	102	99	95	53
high	218	210	204	192	175	112

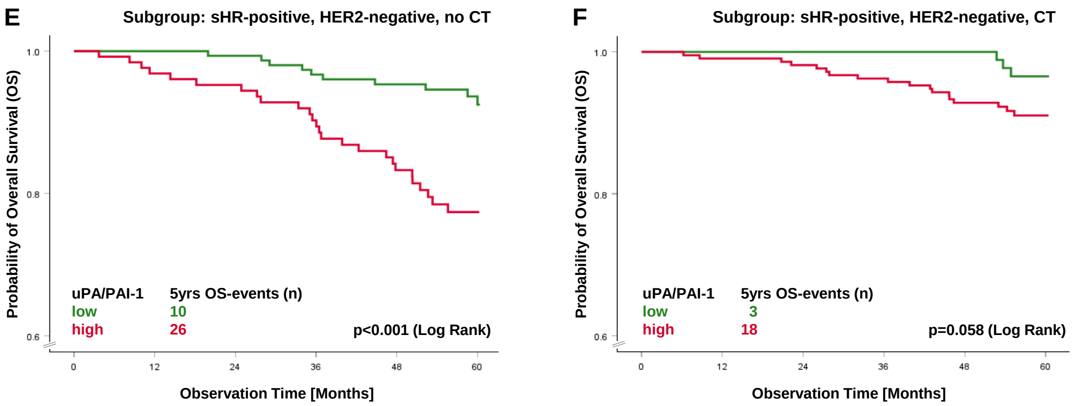

No. at risk	294	282	271	251	228	138
low	165	159	154	145	135	81
high	129	123	117	106	93	57

No. at risk	324	317	312	300	280	169
low	106	104	102	100	97	55
high	218	213	210	200	183	114

Supplementary Fig. S1: Survival estimates for uPA/PAI-1 status with regard to RFI and OS. The tables present the effective sample size for each interval (numbers at risk).
A, B: sHR positive/HER2 negative patients (n=618), RFI (A) and OS (B)
C, D: RFI for patients with sHR positive/HER2 negative tumours, without adjuvant chemotherapy (C) and treated with chemotherapy (D)
E, F: OS for patients with sHR positive/HER2 negative tumours, without adjuvant chemotherapy (E) and treated with chemotherapy (F)

Abbreviations: *uPA* urokinase-type plasminogen activator, *PAI-1* plasminogen activator inhibitor type 1, *sHR* steroid hormone receptor, *HER2* human epidermal growth factor receptor 2, *CT* chemotherapy

A

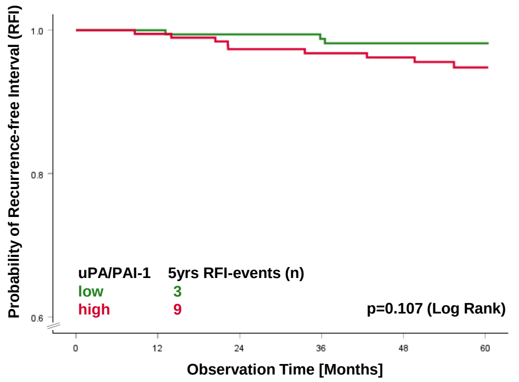

No. at risk	378	366	350	327	306	183
low	181	174	168	159	149	84
high	197	192	182	168	157	99

B

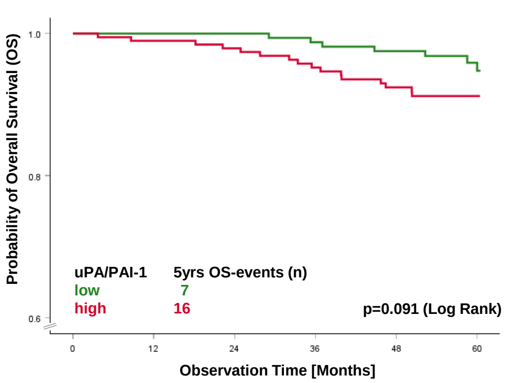

No. at risk	378	366	355	334	311	186
low	181	174	169	161	151	85
high	197	192	186	173	160	101

C

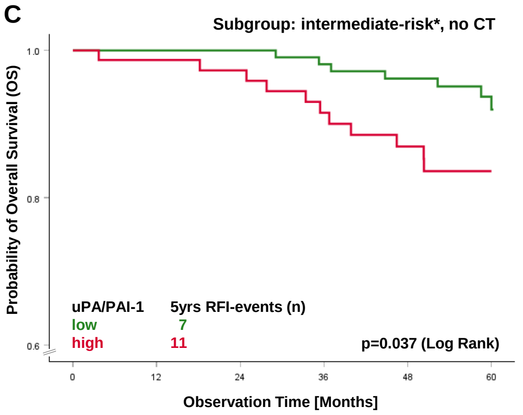

No. at risk	197	188	180	166	150	88
low	120	114	111	104	95	54
high	77	74	69	62	55	34

D

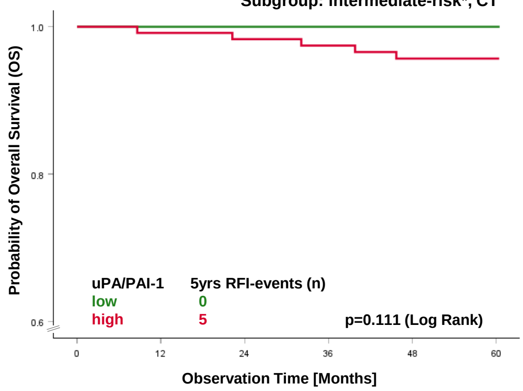

No. at risk	181	178	175	168	161	98
low	61	60	58	57	56	31
high	120	118	117	111	105	67

Supplementary Fig. S2: Survival estimates for uPA/PAI-1 status with regard to RFI and OS. The tables present the effective sample size for each interval (numbers at risk)
A, B: RFI (A) and OS (B) in intermediate-risk group (n=378)
C, D: OS in intermediate-risk group (n=378), without adjuvant chemotherapy (C) and treated with chemotherapy (D)

Abbreviations: *uPA* urokinase-type plasminogen activator, *PAI-1* plasminogen activator inhibitor type 1, *sHR* steroid hormone receptor, *HER2* human epidermal growth factor receptor 2, *CT* chemotherapy
*≥ 35yrs, ≤ pN1, G2, sHR-positive/HER2-negative

Patient groups	Number of patients [n]	Number of RFI-events [n]	Hazard ratio (HR) (95% CI)	p-value
**Total uPA/PAI-1-cohort**	813	70	**2.60** (1.293-5.227)	<0.001
Age ≤ 50 years	195	19	**3.47** (1.009-11.886)	**0.048**
Age > 50 years	618	51	**4.05** (1.823-8.994)	**<0.001**
Tumour size ≤ 2cm	418	20	**14.43** (1.920-108.468)	**0.009**
Tumour size > 2cm	395	50	**2.71** (1.316-5.572)	**0.007**
Nodal status pN0 + pN1	729	35	**3.70** (1.638-8.218)	**0.002**
Nodal status pN0	497	28	**3.95** (1.369-11.376)	**0.011**
Nodal status pN1	232	17	3.16 (0.908-10.994)	0.071
Nodal status ≥ pN2	84	25	2.76 (0.826-9.243)	0.099
Grading G1 + G2	600	36	**2.86** (1.304-6.278)	**0.009**
Grading G3	213	34	**4.31** (1.032-17.978)	**0.012**
sHR-negative	118	29	4.52 (0.614-33.271)	0.139
sHR-positive	695	41	**2.68** (1.282-5.630)	**0.009**
HER2-negative	699	54	**3.75** (1.831-7.688)	**<0.001**
HER2-positive	114	16	3.47 (0.458-26.245)	0.229
sHR-positive/ HER2-negative, pN0	385	15	**3.49** (0.98412.369)	**0.050**

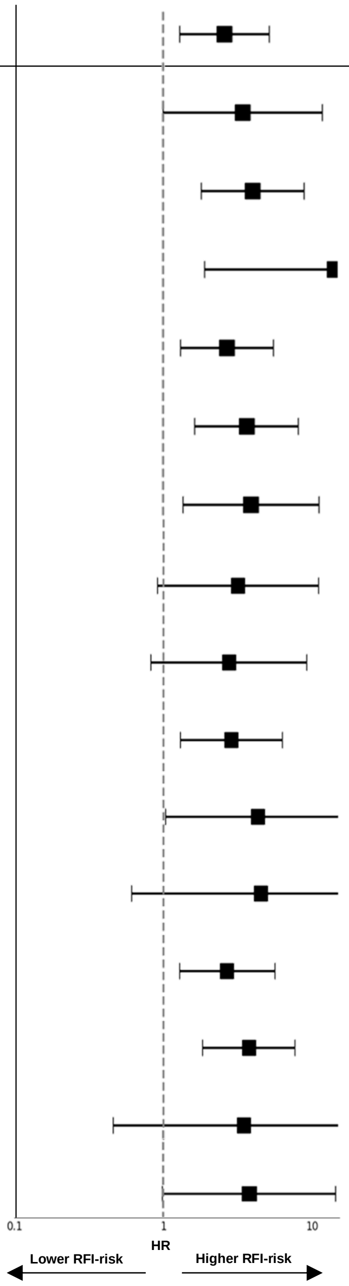

Supplementary Fig. S3: Multivariable analyses comparing high uPA/PAI-1 status to low uPA/PAI-1 status in the total uPA/PAI-1-cohort and in selected subgroups concerning 5-years disease-related events (RFI)

Abbreviations: *uPA* urokinase-type plasminogen activator, *PAI-1* plasminogen activator inhibitor type 1, *sHR* steroid hormone receptor, *HER2* human epidermal growth factor receptor 2, *CI* confidence interval
bold: p-value < 0.05

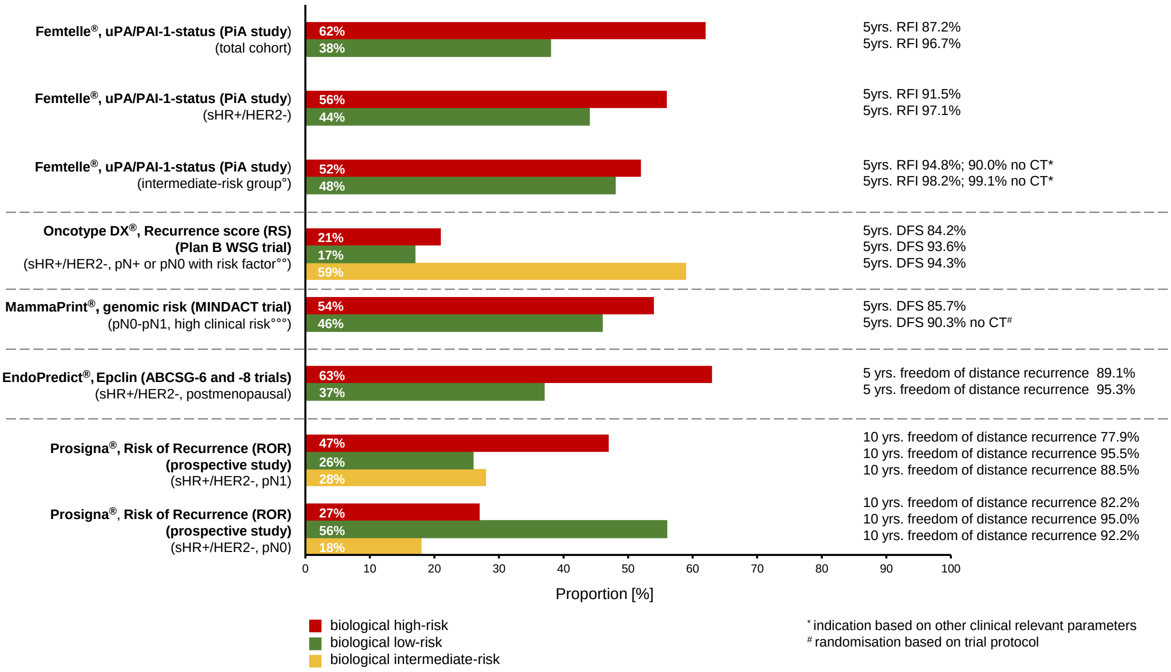

Supplementary Fig. S4: Proportion of groups by different biological risk assessments and clinical outcome using uPA/PAI-1 status and gene expression profiling (GEPs)

Abbreviations: *uPA* urokinase-type plasminogen activator, *PAI-1* plasminogen activator inhibitor type 1, *sHR* steroid hormone receptor, *HER2* human epidermal growth factor receptor 2, *CT* chemotherapy

° ≥35yrs, ≤pN1, G2, sHR positive/HER2 negative
°° ≥pT2, grade 2/3, high uPA/PAI-1, <35 years, or HR negative
°°°clinical risk stratification by using a modified version of Adjuvant! Online

Supplementary Table S1: Patients characteristics and histopathological parameters of the uPA/PAI-1-cohort compared to total cohort

**Characteristics**	**Total cohort**		**uPA/PAI-1-cohort**	
	**1,270**	**(%)**	**813**	**(%)**
**Age in years***				
< 35	33	(2.6)	15	(1.9)
35-50	311	(24.5)	180	(22.1)
> 50	926	(72.9)	618	(76.0)
**Tumour histology***				
Ductal (NST)	636	(81.8)	648	(79.5)
Lobular	528	(13.5)	122	(16.2)
Others	106	(4.7)	43	(4.3)
**Tumour size in cm**				
≤ 2	636	(50.1)	418	(51.4)
> 2	634	(49.9)	395	(48.6)
**Nodal status***				
pN0	780	(61.4)	497	(61.1)
pN1	283	(22.3)	232	(28.5)
≥ pN2	207	(16.3)	84	(10.4)
**Grading***				
G1	167	(13.2)	93	(11.4)
G2	794	(62.5)	507	(62.4)
G3	309	(24.3)	213	(26.2)
**ER status***				
Positive (≥ 1%)	210	(80.3)	687	(84.5)
Negative (< 1%)	1060	(19.7)	126	(15.5)
**PgR status***				
Positive (≥ 1%)	862	(67.9)	572	(70.4)
Negative (< 1%)	408	(32.1)	241	(29.6)
**sHR status***				
Positive (ER and/or PgR ≥ 1%)	1038	(81.7)	695	(85.5)
Negative (ER and PgR < 1%)	232	(18.3)	118	(14.5)
**HER2 status***				
Positive (DAKO 2 if ISH positive, DAKO 3)	210	(16.5)	114	(13.9)
Negative (DAKO 0, 1 or 2 if ISH negative)	1060	(83.5)	699	(86.1)
**IHC-groups***				
sHR-positive and HER2-negative	907	(71.4)	618	(76.0)
sHR-positive and HER2-positive	133	(10.5)	79	(9.7)
sHR-negative and HER2-positive	78	(6.1)	35	(4.3)
TNBC	152	(12.0)	81	(10.0)

Abbreviations: *uPA* urokinase-type plasminogen activator, *PAI-1* plasminogen activator inhibitor type 1, *ER* estrogen receptor, *PgR* progesterone receptor, *SHR* steroid hormone receptor, *HER2* human epidermal growth factor receptor 2, *IHC* Immunohistochemistry, *ISH* in-situ hybridization, *TNBC* triple-negative breast cancer
*p-value (Pearson χ^2 test) < 0.05

Supplementary Table S2: Proportion of low and high uPA/PAI-1 status in selected groups

Characteristics	uPA/PAI-1-cohort<br>n	uPA/PAI-1 low<br>n (%)	uPA/PAI-1 high<br>n (%)
	813	306 (38.0)	507 (62.0)
**Age in years***			
< 35	15	4 (26.7)	11 (73.3)
35-50	180	71 (39.4)	109 (60.6)
> 50	618	231 (37.4)	387 (62.6)
**Tumour histology***			
Ductal (NST)	648	221 (34.1)	427 (65.9)
Lobular	122	72 (59.0)	50 (41.0)
Others	43	13 (30.2)	30 (69.8)
**Tumour status***			
≤ pT1	418	164 (39.2)	254 (60.8)
> pT2	395	142 (35.9)	253 (64.1)
**Nodal status***			
pN0	497	196 (39.4)	301 (60.6)
pN1	232	90 (38.8)	142 (61.2)
≥ pN2	84	20 (23.8)	64 (76.2)
**Grading`***			
G1	93	43 (45.2)	50 (54.8)
G2	507	221 (43.6)	286 (56.4)
G3	213	42 (19.7)	171 (80.3)
**sHR status***			
Positive (ER and/or PgR ≥ 1%)	696	290 (41.7)	406 (58.3)
Negative (ER and PgR < 1%)	117	16 (13.7)	101 (86.3)
**HER2 status***			
Negative (DAKO 0, 1 or 2 if ISH negative)	699	286 (40.9)	413 (59.1)
Positive (DAKO 3, DAKO 2 if ISH positive)	114	20 (17.7)	94 (82.3)
**IHC-groups***			
sHR-positive and HER2-negative	618	271 (43.9)	347 (56.1)
sHR-positive and HER2-positive	78	19 (24.4)	59 (75.6)
sHR-negative and HER2-positive	36	1 (2.9)	35 (97.1)
TNBC	81	15 (18.5)	66 (81.5)

Abbreviations: *uPA* urokinase-type plasminogen activator, *PAI-1* plasminogen activator inhibitor type 1, *ER* estrogen receptor, *PgR* progesterone receptor, *sHR* steroid hormone receptor, *HER2* human epidermal growth factor receptor 2, *IHC* Immunohistochemistry, *ISH* in-situ hybridization, *TNBC* triple-negative breast cancer

*p-value (Pearson χ^2 test) < 0.05

Supplementary Table S3: Association of low and high uPA/PAI-1 status with selected subgroups

Characteristics	uPA/PAI-1 low				uPA/PAI-1 high			
	Sample size n=306	Odds ratio	95% CI	p-value	Sample size n=507	Odds ratio	95% CI	p-value
**Age in years**								
< 35	4	Ref			11	Ref		
35-50	71	1.79	0.549-5.846	0.334	109	0.56	0.171-1,822	0.334
> 50	231	1.64	0.517-5.215	0.401	387	0.61	0.192-1.935	0.401
**Tumour histology**								
Ductal (NST)	221	Ref			427	Ref		
Lobular	72	**2.78**	1.873-4.133	**<0.001**	50	**0.36**	0.242-0.534	**<0.001**
Others	13	0.84	0.428-1.637	0.604	30	1.19	0.611-2.336	0.604
**Tumour size in cm**								
< 2	164	Ref			254	Ref		
2-5	122	0.79	0.590-1.060	0.117	239	1.27	0.943-1.696	0.117
≥ 5	20	**2.21**	1.087-4.503	**0.029**	14	**0.45**	0.222-0.920	**0.029**
**Nodal status**								
pN0 and pN1	286	Ref			443	Ref		
≥ pN2	20	**0.48**	0.287-0.817	**0.007**	64	**2.07**	1.224-3.488	**0.007**
**Grading**								
G1	43	Ref			50	Ref		
G2	221	0.90	0.576-1.400	0.637	286	1.11	0.714-1.735	0.637
G3	42	**0.29**	0.168-0.485	**<0.001**	171	**3.50**	2.063-5.944	**<0.001**
**sHR status**								
Positive (ER and/or PgR ≥ 1%)	290	**4.57**	2.939-7.897	**<0.001**	405	**0.22**	0.127-0.379	**<0.001**
Negative (ER and PgR < 1%)	16	Ref			102	Ref		
**HER2 status**								
Positive (DAKO 2 if ISH positive, DAKO 3)	20	Ref			94	Ref		
Negative (DAKO 0, 1 or 2 if ISH negative)	286	**3.21**	1.937-5.328	**<0.001**	413	**0.31**	0.188-0.516	**<0.001**
**IHC-groups**								
sHR-positive and HER2-negative	271	Ref			347	Ref		
HER2-positive any sHR	20	**0.27**	0.164-0.453	**<0.001**	94	**3.67**	2.208-6.101	**<0.001**
TNBC	15	**0.29**	0.162-0.521	**<0.001**	66	**3.44**	1.919-6.154	**<0.001**

Abbreviations: *uPA* urokinase-type plasminogen activator, *PAI-1* plasminogen activator inhibitor type 1, *sHR* steroid hormone receptor, *HER2* human epidermal growth factor receptor 2, *IHC* Immunohistochemistry, *ISH* in-situ hybridization, *TNBC* triple-negative breast cancer, *CI* confidence interval
bold: p-value < 0.05

Supplementary Table S4: Univariable and multivariable analyses of uPA/PAI-1 status with regard to OS for selected characteristics (5 years)

Characteristics	Overall survival, 5 years (97 events)							
			Univariable analysis			Multivariable analysis		
	Sample size	Events	Hazard ratio	95% CI	p-value	Hazard ratio	95% CI	p-value
**uPA/PAI-1-status**								
low	306	17	1			1		
high	507	80	**2.99**	1.774-5.054	**<0.001**	**2.38**	1.359-4.156	**0.002**
**Age in years**								
< 35	15	1	1					
35-50	180	12	0.99	0.129-7.630	0.994			
> 50	618	84	2.15	0.300-15.476	0.445			
**Tumour size in cm**								
≤ 2	418	24	1			1		
> 2	395	73	**3.42**	2.158-5.430	**<0.001**	**2.44**	1.501-3.956	**<0.001**
**Nodal status**								
pN0	497	40	1					
pN1	232	28	1.50	0.926-2.343	0.099			
≥ pN2	84	29	**4.80**	2.958-7.779	**<0.001**			
**Nodal status**								
pN0 and pN1	729	68	1			1		
≥ pN2	84	29	**1.50**	0.926-2.433	0.099	**2.54**	1.595-4.036	**<0.001**
**Grading**								
G1	93	5	1			1		
G2	507	48	1.96	0.780-4.923	0.152	1.53	0.605-3.880	0.367
G3	213	44	**4.39**	**1.738-11.060**	**0.002**	2.24	0.849-5.905	0.103
**sHR status**								
Positive (ER and/or PgR ≥ 1%)	695	70	1					
Negative (ER and PgR < 1%)	118	27	**2.47**	1.583-3.849	**<0.001**			
**HER2 status**								
Positive (DAKO 2 if ISH positive, DAKO 3)	114	20	**1.65**	1.007-2.694	**0.047**			
Negative (DAKO 0, 1 or 2 if ISH negative)	699	77	1					
**IHC-groups**								
sHR-positive and HER2-negative	618	57	1			1		
HER2-positive any sHR	114	20	**1.96**	1.1776-3.259	**0.01**	1.20	0.700-2.069	0.503
TNBC	81	20	**2.86**	1.720-4.764	**<0.001**	1.58	0.899-2.779	0.112

Abbreviations: *uPA* urokinase-type plasminogen activator, *PAI-1* plasminogen activator inhibitor type 1, *sHR* steroid hormone receptor*, HER2* human epidermal growth factor receptor 2*, IHC* Immunohistochemistry*,* ISH in-situ hybridization, *TNBC* triple-negative breast cancer, *CI* confidence interval
bold: p-value < 0.05

Supplementary Table S5: Probabilities of disease-related events or any death with regard to uPA/PAI-1 status in selected subgroups

Patient groups	Recurrence-free Interval, 5 years				Oversall Survival, 5 years			
	Sample size	Events	[%]	95% CI	Sample size	Events	[%]	95% CI
**Total uPA/PAI-1-cohort**								
uPA/PAI-1 low	306	10	96.7	94.5-98.9	306	17	93.9	91.0-96.8
uPA/PAI-1 high	507	60	87.2	84.1-90.3	507	89	82.9	79.4-86.4
**sHR-positive/HER2-negative**								
uPA/PAI-1 low	271	7	97.1	94.0->99.9	271	13	94.1	91.0-97.2
uPA/PAI-1 high	347	26	91.5	89.3-93.7	347	44	86.1	82.2-90.0
**sHR-positive/HER2-negative, no CT**								
uPA/PAI-1 low	165	4	98.1	95.9->99.9	165	10	92.5	88.0-97.0
uPA/PAI-1 high	129	11	89.9	83.6-96.2	129	26	77.4	69.8-85.0
**sHR-positive/HER2-negative, CT**								
uPA/PAI-1 low	106	4	95.7	91.6-99.8	106	3	96.6	92.7->99.9
uPA/PAI-1 high	218	15	92.7	89.2-96.2	218	18	91.0	87.1-94.9
**Intermediate-risk***								
uPA/PAI-1 low	181	4	98.2	96.2->99.9	181	7	94.8	90.9-98.7
uPA/PAI-1 high	197	9	94.8	91.5-98.1	197	16	91.2	87.1-95.3
**Intermediate-risk, no CT ***								
uPA/PAI-1 low	120	2	99.1	97.3->99.9	120	7	92.0	86.1-97.9
uPA/PAI-1 high	77	6	90.0	82.2-97.8	77	11	83.5	74.5-92.5
**Intermediate-risk, CT***								
uPA/PAI-1 low	61	2	96.5	91.8->99.9	61	0		
uPA/PAI-1 high	129	3	97.4	94.5->99.9	129	5	95.7	91.6-99.8

Abbreviations: *uPA* urokinase-type plasminogen activator, *PAI-1* plasminogen activator inhibitor type 1, *sHR* steroid hormone receptor, *HER2* human epidermal growth factor receptor 2, *CT* chemotherapy, *CI* confidence interval

* ≥ 35yrs, ≤ pN1, G2, sHR positive/HER2 negative

Supplementary Table S6: Impact of uPA/PAI-1 status with regard to OS in selected subgroups

Patient groups	Overall Survival, 5 years			
	Sampe size	Events	Hazard ratio	95% CI
**Total uPA/PAI-1-cohort**				
uPA/PAI-1 low	306	17		
uPA/PAI-1 high	507	80	**2.38**	1.359-4.156
**sHR-positive/HER2-negative**				
uPA/PAI-1 low	271	13		
uPA/PAI-1 high	347	44	**2.77**	1.489-5.134
**sHR-positive/HER2-negative, no CT**				
uPA/PAI-1 low	165	10		
uPA/PAI-1 high	129	26	**3.61**	1.741-7.492
**sHR-positive/HER2-negative, CT**				
uPA/PAI-1 low	106	3		
uPA/PAI-1 high	218	18	3.08	0.906-10.449
**Intermediate-risk***				
uPA/PAI-1 low	181	2		
uPA/PAI-1 high	197	16	2.12	0.873-5.156
**Intermediate-risk, no CT ***				
uPA/PAI-1 low	120	7		
uPA/PAI-1 high	77	11	**2.66**	1.032-6.871
**Intermediate-risk, CT***				
uPA/PAI-1 low	61	0		
uPA/PAI-1 high	129	5		

Abbreviations: *uPA* urokinase-type plasminogen activator, *PAI-1* plasminogen activator inhibitor type 1, *sHR* steroid hormone receptor, *HER2* human epidermal growth factor receptor 2, *CT* chemotherapy, *CI* confidence interval

* ≥ 35yrs, ≤ pN1, G2, sHR positive/HER2 negative

bold: p-value < 0.05
